# Supplementary figures and images for: Prevalence of poor glycemic control and the monitoring utility of glycated albumin among diabetic patients attending clinic in tertiary hospitals in Dodoma, Tanzania: A cross-sectional study protocol
Source: PLoS One. 2024 Sep 4;19(9):e0289388. doi: 10.1371/journal.pone.0289388 (PMC11373853; doi:10.1371/journal.pone.0289388)

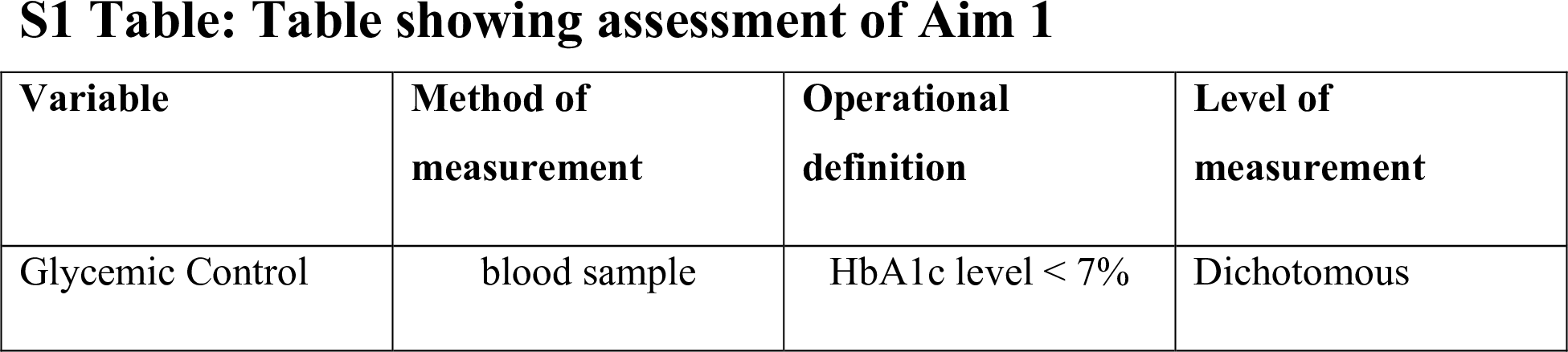

Supplement: S1 Table — (TIF) [file pone.0289388.s001.tif]

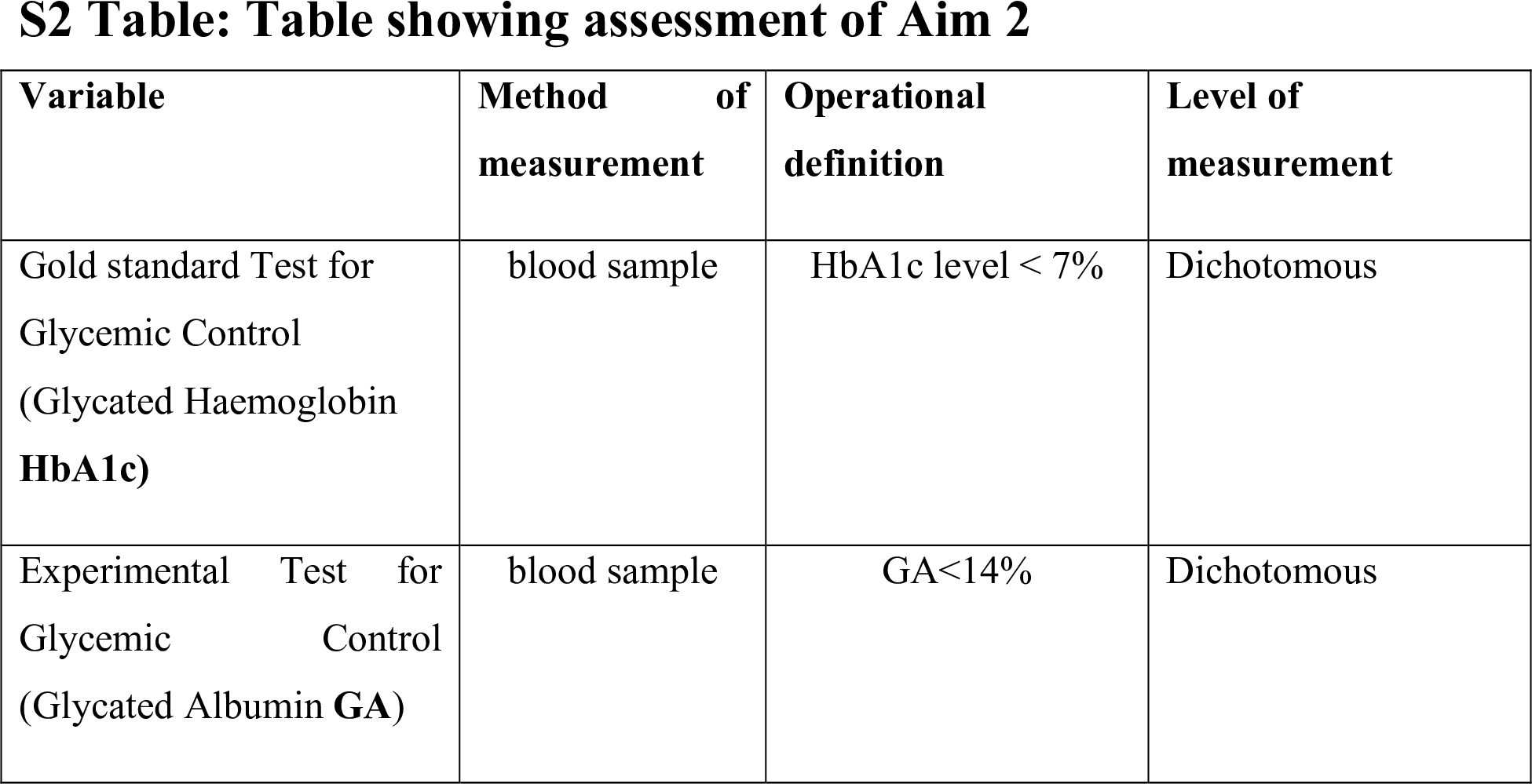

Supplement: S2 Table — (TIF) [file pone.0289388.s002.tif]
