# Supplementary material for: Prevalence of poor glycemic control and the monitoring utility of glycated albumin among diabetic patients attending clinic in tertiary hospitals in Dodoma, Tanzania: A cross-sectional study protocol
Source: PLoS One. 2024 Sep 4;19(9):e0289388. doi: 10.1371/journal.pone.0289388 (PMC11373853; doi:10.1371/journal.pone.0289388)
Supplement: Attachment — Submitted filename: Response to Reviewers.pdf [file pone.0289388.s003.pdf]

DR. GEORGE GABRIEL MKUMBI

P O Box 259 Dodoma

1 TIBA STREET,

41218 IYUMBU, DODOMA

TANZANIA.

Email: mkumbig@gmail.com

11/11/2023.

EDITOR-IN-CHIEF PLOS ONE

1265 BATTERY STREET, SUITE 200

SAN FRANCISCO, CA 94111

UNITED STATES

DEAR EDITOR,

RE: RESUBMISSION OF A STUDY PROTOCOL TITLED - "PREVALENCE OF POOR GLYCEMIC CONTROL AND THE MONITORING UTILITY OF GLYCATED ALBUMIN AMONG DIABETIC PATIENTS ATTENDING CLINIC IN TERTIARY HOSPITALS IN DODOMA, TANZANIA: A CROSS-SECTIONAL STUDY PROTOCOL" [PONE-D-23-20966]

I hope this letter finds you in good health and high spirits. I am writing to submit a revised version of my manuscript titled above for consideration. I am grateful for the opportunity to address the valuable feedback provided by the reviewers, and I believe that the revisions made have significantly improved the quality and rigour of the work.

I want to express my sincere gratitude to you and the reviewers for dedicating your time and expertise to evaluate my manuscript. Your insightful comments and suggestions have been instrumental in refining the content and methodology. I have carefully considered each comment and have made the necessary revisions, ensuring that the manuscript now adheres to the high standards set by PLoS ONE.

Specifically, I would like to highlight the following fundamental changes that have been made:

1. **I have revised the title to enhance clarity to read as written in the subject of this letter.**
2. Table 1 to 4 below Details the significant revisions and how they address the reviewers' comments.

Change matrix following reviewers' comments.

Table 1: Sections Comments

S/n	SECTIONS	Comments	Response
1.	Introduction	• Add a section describing the prevalence of poor glycemic control in Tanzania	• The current information on prevalence was added
2.	Sample size estimation	• Can the authors provide any justification for using the cited reference from Uganda, with a prevalence of 84.3%? There are a number of published studies conducted in Tanzania with a lower prevalence of poor glycemic control, which would be a better choice than the one used.	• This was the most current data on prevalence in the East African region. It was done in a university teaching hospital similar to the study setting. I have come across a study on prevalence from Ifakara done in 2016 in a rural population; I felt this was old, and its population differs from my area, which consists of a mixed population. Other studies have been done on the subject. The latest was in 2014 from the country's national hospital and major hospitals in Dar es Salaam, meaning a more urbanised population. The study I have chosen suits my purpose, being up-to-date and not from major hospitals and serving a similar population like the one I am to study.
3.	Exclusion and Inclusion criteria	• How will you identify these patients? "Patients who are at risk of receiving or donating blood"	• This error was noted and removed
		• Will the study not consider specific types of diabetic patients? E.g Type 1, type 2, autoimmune diabetes e.t.c?	• This is a cross-sectional study with a major aim to determine the utility of a test in detecting poor glycemic control and how prevalent it is in our clinical settings. Addressing specific types of diabetes categories is out of scope for now, as it will require a robust methodology to diagnose and confirm such categories, and it is deemed difficult in the study settings.
4.	Clinical examination	• The names, brands and models, and city of manufacture of the equipment and machines should be written in brackets. For example, you write: "A blood pressure (BP) reading will be taken using an automated digital machine (AD Medical Instruments, Beijing, China)." Revise this throughout the manuscript.	• Revision of the said script was made accordingly.

5.	Data collection methods.	• Revise the dates for the commencement of data collection and pilot study	• Dates revisions were made to accommodate 4 months for data collection and 2 months for analysis and write-up
		• In this section, it is stated that BP will be measured using a Mercury sphygmomanometer, while in contrast to the previous section (clinical examination), it is clearly explained that BP will be gauged using an automated digital BP machine. To make it clear, why is this so?	• Revision was made in the section, and the more accurate mercury BP machine will be used. Both sections of the documents read as a mercury BP machine will be used.
6.	Independent variables	• Revise the paragraph describing Glycemic control. what you describe is not related to the description of glycemic control as the independent variable. The paragraph is poorly organised, incoherent and congested with unnecessary explanations.	• The paragraph was improved for clarity and coherence. The variables were revised.
7.	Ethical issues	• Give a concise elaboration. Don't repeat explanations from the methodology sections.	• Section was revised to remove repetition and shortened

8.	Discussion	• Provide a reference for the use of HbA1c a Gold standard method. A number of studies recommend OGTT as the Gold standard method.	• The **glycated hemoglobin (HbA1c)** test is currently considered the gold standard for assessing glycemic control and response to therapy in patients with type 1 and type 2 diabetes[2]. The HbA1c test measures the average blood glucose level over the past 2-3 months [1]. The American Diabetes Association recommends that glycemic status be assessed at least two times a year in patients who are meeting treatment goals and who have stable glycemic control and at least quarterly and as needed in patients whose therapy has recently changed and/or who are not meeting glycemic goals [1].<br><br>• reference<br>(1) Glycemic variability and cardiovascular disease in patients with type 2 .... https://drc.bmj.com/content/9/1/e002032.<br>(2) 6. Glycemic Targets: Standards of Medical Care in Diabetes—2021. https://diabetesjournals.org/care/article/44/Supplement_1/S73/30909/6-Glycemic-Targets-Standards-of-Medical-Care-in.<br>(3) RACGP - Glucose monitoring. https://www.racgp.org.au/clinical-resources/clinical-guidelines/key-racgp-guidelines/view-all-racgp-guidelines/diabetes/glucose-monitoring.<br>(4) Target for Glycemic Control: - American Diabetes Association. https://diabetesjournals.org/care/article/32/suppl_2/S199/26598/Target-for-Glycemic-ControlConcentrating-on.
9.	Dissemination of the findings	• The statement “different peer-review journals for publication” should be revised. Duplicate or Salami publication is unethical in research. Manuscript should be submitted to only one peer-reviewed journal. After the previous submission's editorial decision has been made, resubmissions are permitted.	• Corrections were made in response to this concern accordingly.

Table 2: Editor Comments

S/n	Comments	Response
1.	A range of statistical techniques have been listed, indicating a comprehensive plan to thoroughly analyze the data. However, the authors have mentioned calculating a percentage for demographic variables (e.g., gender, place of residence). They should be cautious with smaller sample sizes as percentages may not provide a true representation. Actual counts or proportions might be more informative in such cases.	• This is dully noted, and taken into consideration.
2.	The inclusion of Cronbach's alpha to assess data reliability is a strong addition.	• This is dully noted
3.	The research plan has clearly laid out the ethical considerations and approvals needed. It's good to see the references to consent and the process for withdrawing from the study. For completeness and clarity, the authors should consider having a plan to handle any adverse events or complications that might arise from the blood draw.	• Adverse events or complications that might arise from the blood draw are addressed in the laboratory section of the document.<br>• consent and the process for withdrawing from the study was addressed under ethical consideration section, which is refined for clarity.
4.	The timeline is straightforward. However, depending on the sample size, two months for data collection and only one month for analysis might be tight, especially if unforeseen issues arise. If that is the case, the authors can consider amending their protocol based on realistic timelines and add in the protocol some of the comments raised here.	• Dates revisions were made to accommodate 4 months for data collection and 2 months for analysis and write-up

5.	The comparison of GA and HbA1c is an essential part of this work. Ensure that the benefits and limitations of each are clearly demarcated and not mixed. Additionally, while the authors have addressed the clinical implications of HbA1c and GA, it may be worth briefly mentioning any cost or accessibility differences between the two, if relevant.	• The 3rd paragraph of the introduction explains the weakness and strength of HbA1c, followed by two paragraphs on GA and a final concluding paragraph of the discussion summarizes the comparisons. I have not found published data on costs of the test.
6.	It's commendable that they have addressed potential study limitations, but consideration to adding a dedicated "Limitations" section will improve clarity.	• Thank you for this wonderful input; however, we feel that the limitations are best to be described in the discussion section as it was done.
7.	The conclusion neatly sums up the relevance of the study. However, considerations to add potential implications for policy or clinical practice based on expected findings will add value.	• This is a wonderful suggestion that we also thought about, but however owing to the small nature of the study and it being a cross-sectional study, adding a note on its implications on policymaking or change will be an oversell and unrealistic; however, the study lays a foundation for conducting robust studies that will have the said implication. This is discussed as such throughout the text.
8.	The conclusion mentions the "current gold standard marker." However, a reader unfamiliar with the topic might benefit from clarifying that HbA1c is that standard marker.	• This is dully noted and amendments were made.
9.	Consider adding a statement about the potential for presenting findings at conferences or symposiums, if applicable.	• Corrections were made in response to this concern accordingly.
10.	While the hospitals and participants have been acknowledged the authors should also consider acknowledging any funding or grants that supported this work, if applicable.	• Corrections were made in response to this concern accordingly.
11.	Consider registering the study to one of clinical research data bases like clinical trial.gov	• This is dully noted

12.	Lastly, they should ensure a thorough proofreading for grammatical and typographical errors before the final submission.	• Grammatic corrections and proof reading was done to improve the overall quality of the work.

Table 3: Reviewer #1 Comments

S/n	Comments	Response
1.	The manuscript is written in the future tense while the study has already been conducted, additionally the manuscript reads like a research proposal.	• This is a study protocol for a study the is currently on going and this document was submitted in July before commencement of the study itself.
2.	Although there is a discussion section there are no results to be discussed.	• The study has not provided any results at the moment, as its in the data collection stage data analysis is not made.
3.	Manuscript arrangement needs to follow standard journal format	• The manuscript was corrected to reflect the standard format of the journal.
4.	Results and conclusion seem to be missing from abstract	• The study has not provided any results at the moment, as its in the data collection stage
5.	Key references are needed, for example: "Additionally, it is suitable for individuals' undergoing hemodialysis and its levels are unaffected by anaemia or haemolytic processes. GA is preferable to fructosamine because it is not impacted differently by different serum proteins"	• Revisions and additions were made

Table 4: Reviewer #2 Comments

S/n	Comments	Response
1.	I would advise thoroughly checking any typos and grammatical problems throughout the study protocol. This will make the proposal more comprehensible and professional-looking. For instance, glycosylated and glycated hemoglobin are mixed in various locations.	• Grammatic corrections and proof reading was done to improve the overall quality of the work.
2.	There are some repetitions of similar explanations in the paragraphs. This repetition can be confusing for the reader and can make the study protocol not appealing and seem less polished. Authors should thoroughly revise the whole document and make sure they remove/paraphrase wordy sentences and descriptions recurring in different sections. For example, The explanations about data collection by the use of questionnaires have been repeated several times in the text.	• This is dully noted, and the work is rewritten to remove repetition, wordy sentences and phrases were removed /rephrased.
3.	Why were other factors that may affect glycemic control like physical activity, and diet not considered.?	• Inclusion of such mentioned factors is out of scope and aim of our study. Our aim to determine the prevalence and utility of GA as a biomarker.
4.	Authors should define all abbreviations the first time they are used. Also, abbreviations should be used consistently throughout the study proposal.	• Dully noted and revisions were made to improve the overall consistency of the work.

5.	Under the Discussion section, authors should revise thoroughly and provide a reference for the applicability of cross-sectional studies in studying the Validity of diagnostic tests.	• Most of the studies including the meta-analysis by Chume et,al. All featured across sectional study designs in study of the validity of the biomarker and are referenced in multiple sections throughout the text including the discussion.

I believe these revisions have significantly strengthened the manuscript and addressed the concerns the reviewers raised. I am confident that the revised version aligns well with the scope and standards of PLoS ONE and will contribute meaningfully to the field.

Enclosed with this letter is the revised manuscript and a marked-up copy highlighting the changes made in response to the reviewers' comments.

I kindly request your consideration for re-evaluating the revised manuscript for publication. Please do not hesitate to contact me if you require further information or have additional suggestions.

Thank you once again for your time and consideration. I look forward to hearing from you soon.

Sincerely,

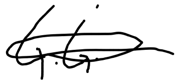

Dr. George Gabriel Mkumbi, MD

Corresponding and first Author
