# Supplementary material for: Prevalence of poor glycemic control and the monitoring utility of glycated albumin among diabetic patients attending clinic in tertiary hospitals in Dodoma, Tanzania: A cross-sectional study protocol
Source: PLoS One. 2024 Sep 4;19(9):e0289388. doi: 10.1371/journal.pone.0289388 (PMC11373853; doi:10.1371/journal.pone.0289388)
Supplement: Attachment — Submitted filename: Response to Reviweres.docx [file pone.0289388.s005.docx]

DR. GEORGE GABRIEL MKUMBI

P O Box 259 Dodoma 1 TIBA STREET,

41218 IYUMBU, DODOMA TANZANIA.

Email: [mkumbig@gmail.com](mailto:mkumbig@gmail.com)

**21/03/2024.**

EDITOR-IN-CHIEF PLOS ONE

1265 BATTERY STREET,

SUITE 200 SAN FRANCISCO,

CA 94111 UNITED STATES

DEAR EDITOR,

**RE: THE THIRD RESUBMISSION OF A STUDY PROTOCOL TITLED - "PREVALENCE OF POOR GLYCEMIC CONTROL AND THE MONITORING UTILITY OF GLYCATED ALBUMIN AMONG DIABETIC PATIENTS ATTENDING CLINIC IN TERTIARY HOSPITALS IN DODOMA, TANZANIA: A CROSS-SECTIONAL STUDY PROTOCOL" [PONE-D-23-20966R2]**

I hope this letter finds you in good health and high spirits. I am writing to submit a third revised version of my manuscript titled above for consideration. This resubmission is in response to the invaluable feedback provided by the reviewers, and I would like to thank the reviewers and the editorial team for their time and constructive comments on my work.

Specifically, I would like to highlight the following fundamental changes that have been made as summarised in the table below:

**Change matrix following reviewers’ comments.**

| **S/n** | **Comments** | **Response** |
| --- | --- | --- |
| 1. | - We note that the grant information you provided in the ‘Funding Information’ and ‘Financial Disclosure’ sections do not match.      - When you resubmit, please ensure that you provide the correct grant numbers for the awards you received for your study in the ‘Funding Information’ section. | - Thank you for noting this inconsistency, the explanation for this discrepancy was asked and addressed in my first resubmission but unfortunately, amendments were regrettably overlooked. - The funders did not sponsor the study, but the residency program that I was on during the time of the study conception. - Our updated financial disclosure I would like it to read as “The authors received no specific funding for this work” |
| 2. | - Thank you for noting that your data will be made available. Please note that PLOS ONE’s policies on Data Availability require that all data necessary to replicate the findings of the manuscript be made available upon acceptance. - PLOS ONE strongly recommends that this data be deposited to a stable public repository such as Figshare (http://figshare.com/) or Dryad (datadryad.org). For a list of other PLOS recommended repositories, please visit: http://journals.plos.org/plosone/s/data-availability#loc-recommended-repositories. - Should your paper be accepted for publication, we will ask that you please provide a DOI or accession number for your dataset. We will then update the Data Availability statement associated with your manuscript on your behalf. Ensuring data deposition before acceptance of your manuscript will help to prevent delays in the acceptance process. | - Since this publication is a Study Protocol for a proposed study, no datasets were generated or analysed during the current publication. All relevant data from this study will be made available upon study completion in subsequent publications. |

***Table 1*: Comments**

I believe these revisions have significantly strengthened the manuscript and addressed the concerns the reviewers raised. I am confident that the revised version aligns well with the scope and standards of PLoS ONE.

Enclosed with this letter is the revised manuscript and a marked-up copy highlighting the changes made in response to the reviewers' comments.

I kindly request your consideration for re-evaluating this version of the revised manuscript for publication. Please do not hesitate to contact me if you require further information or have additional suggestions.

Thank you once again for your time and consideration. I look forward to hearing from you soon. Sincerely,

Dr. George Gabriel Mkumbi, MD
