# Supplementary material for: Prevalence of poor glycemic control and the monitoring utility of glycated albumin among diabetic patients attending clinic in tertiary hospitals in Dodoma, Tanzania: A cross-sectional study protocol
Source: PLoS One. 2024 Sep 4;19(9):e0289388. doi: 10.1371/journal.pone.0289388 (PMC11373853; doi:10.1371/journal.pone.0289388)
Supplement: Attachment — Submitted filename: REVIEWER COMMENTS.pdf [file pone.0289388.s004.pdf]

REVIEWER COMMENTS

The authors intend to determine the prevalence of poor glycemic control and the utility of serum glycated albumin (GA) as an index of glycemic control in diabetic patients. The proposed study is important since GA appears to be of great potential in diagnostic efficiency, relatively less costly and the possibility of GA equipment automation. In turn, GA as biomarker for glycemic control will be useful in limited resource settings such as Tanzania.

Nevertheless, the authors need to address the following comments;

1. It is not clear why the authors will screen for eligibility among patients diagnosed with diabetes mellitus for over six months. What was the basis for using 6 months as a cut-f point for eligibility to participate in the study?
2. The authors intend to conduct an interview and self-administer the questionnaire among those would wish to do so. In addition, those wishing to self-administer the question will be given a Swahili version questionnaire. How is this likely to affect the study findings?
3. The authors describe that they will perform physical examinations and/or obtain information such as blood pressure, body mass index and electrocardiogram. However, the relationship between such parameters and the validity of GA in glycemic control is not clearly described in the introduction and methods section such as in the analysis section.
